# We are such stuff / as dreams are made on

**DOI:** 10.3201/eid1708.AC1708

**Published:** 2011-08

**Authors:** Polyxeni Potter

**Affiliations:** Author affiliation: Centers for Disease Control and Prevention, Atlanta, Georgia, USA

**Keywords:** art science connection, emerging infectious diseases, art and medicine, Maxfield Parrish, Masquerade, we are such stuff, as dreams are made of, virus infections, virology, viral replication, about the cover

―William Shakespeare, The Tempest

“We are all doubles… but at night… we meet our sleeper,” wrote Tom Stoppard in his play “Hapgood” (1988). He was approaching a common theme, identity, and how we use disguises to change it. In literature, as in life, we turn the world into a carnival to address personal, social, and cultural objectives. In microbiology carnival de-structuring abounds within the living cells of organisms. And in art, identity and its mysteries and disguises have occupied many, who examine it from all angles, exposing its multifaceted and diverse nature. Maxfield Parrish takes a playful look at it in his own *Masquerade*, on this month’s cover.

“I don’t know what people find or like in me,” Parrish once said. “I’m hopelessly commonplace.”And although he might not have intended it this way, a modest and unassuming man, he had become ubiquitous in his lifetime. In 1925, one in four American households owned one of his prints. His wildly successful calendar of nature scenes alone sold more than a million copies. His box covers for Crane’s Chocolates flew off the shelves. His massive murals, magazine covers, and book illustrations captured the imagination of the public. “As far as the sale of expensive reproductions is concerned, the three most popular artists in the world are Vincent van Gogh, Paul Cézanne, and Maxfield Parrish,” asserted Time magazine in 1936.

A native of Philadelphia, Pennsylvania, Parrish knew none of the financial hardships of the proverbial starving artist. His privileged family recognized and nurtured his talent from early childhood. They traveled to Europe for extended periods to broaden his art horizons and appreciation. In his native state he attended Swarthmore College’s preparatory school; Haverford College, where he studied architecture; the Pennsylvania Academy of Fine Arts; and the Drexel Institute of Art.

His painted dreams, imaginary landscapes, mythical animals, romantic creatures, and classical figures in vivid distinctive hues became fixtures of storybooks and part of the vernacular. All the while he shaped the Golden Age of Illustration and went on to influence Norman Rockwell, who once said about Parrish, “He was one of my Gods.”

Though his long life was not entirely immune to trials, each crisis seemed to propel him to a higher level of greatness. Typhoid fever in his early youth and prolonged convalescence confined him enough to allow etching and drawing lessons from his father, also an artist. And when tuberculosis sent him to Arizona for the hot and dry climate, the Desert Southwest transformed him from illustrator to landscape painter. “I’m done with girls on rocks,” he declared. “I’m quitting my rut now while I’m still able.”

His commercial art ventures gained early national attention. He was interested in the business of art. He created works for the color print market and romantic scenes for a public that craved them. “I’ve always considered myself a popular artist.” His early success enabled him to build a home and studio in Cornish, New Hampshire, setting of his *Land of Make Believe*, where he lived and worked for the rest of his life in the natural environment he loved and recreated in his works. One of his four children, Jean Parrish, became an accomplished artist in her own right.

Parrish embraced use of technology in his work. He had a machine shop in the studio to make his own tools and used photography extensively, often several photographs in one painting. He produced costumes for his fantastic landscape sets. The dramatic palette came from an elaborate technique, secret of the old masters. Over a smooth white ground, he applied several thin layers of transparent paint, each followed by a layer of glaze to achieve luminescence. A certain blue color was named after him, “Parrish blue.”

He devised innovative techniques that anticipated op art, a method of abstraction. Movement, flashing, or other similar optical effects could be achieved by combining photography with geometric shapes to distort figures. Parrish belonged to no formal movement or school. He developed his own unique style. He influenced the likes of Andy Warhol and has been compared to Salvador Dali.

In *The Masquerade*, many Parrish elements come into play. Like many of his paintings, this one has a mixture of fantasy and humor, realism, a dream-like quality, and a generous sprinkling of pageantry and optical illusion. The large red mantle is draped against a repetitive geometric pattern, alternating yellow and black squares that vibrate under direct scrutiny. Otherworldly hues form a natural backdrop of trees, flowers, and leaves.

The mosaic effect of the predominant fabric in this work, along with the carnival disguise, invites a Tom Stoppard interpretation of identity, its changes, and the masquerade. This issue of Emerging Infectious Diseases, with its emphasis on viral infections, provides a tempting overview of de-structuring opportunities in virology. Its own mosaic of viral diseases, the Table of Contents provides an overview of the tiny agents that invade living cells of organisms, spread in many different ways and cause serious illnesses, from gastroenteritis to dengue. Viruses inside living organisms are carnival experts. Their particles enter the host cell and, masquerading as the regular intracellular messengers, subvert the cellular machinery to make new viral components. When the new virus particles are assembled, sometimes with costume elements modified to escape recognition, they leave the cell and “party on” in new host cells. Red cape goes in, checkered robe comes out, until “… our little life / is rounded with a sleep.”

**Figure:**
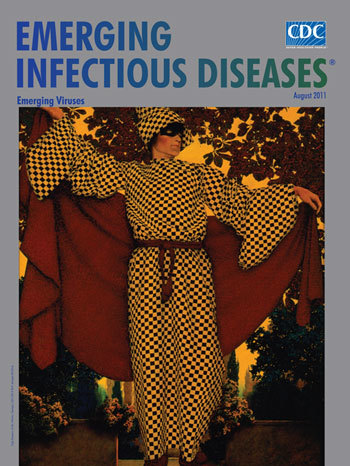
Maxfield Parrish (1870–1966). *Masquerade* (1922) Oil on board (43.2 cm × 35.6 cm). High Museum of Art, Atlanta, Georgia, USA. Gift of Ruth Jernigan McGinty

## References

[1] Amarasinghe A, Kuritsky JN, Letson GW, Margolis HS. Dengue virus infection in Africa. Emerg Infect Dis. 2011;17:1349–54. 21801609 10.3201/eid1708.101515 PMC3381573

[2] Casellas JL-P, Malcolm D, Calle PS, eds. Masquerades; disguise in literature in English from the Middle Ages to the present. Gdańsk (Poland): University of Gdańsk Press; 2004.

[3] Cutler LS, Parrish M, Cutler JG. Maxfield Parrish: a retrospective. San Francisco: Pomegranate Artbooks; 1995.

[4] Coy L. Maxfield Parrish. New York: Watson Guptill; 1973.

[5] Monroe SS. Control and prevention of viral gastroenteritis. Emerg Infect Dis. 2011;17:1347–8. 21801608 10.3201/eid1708.110824 PMC3381538

[6] Smith AG. Maxfield Parrish: master of make-believe. London: Philip Wilson; 2005.

